# Tuning Physicochemical Properties of Boron Nitride-Based Membranes via Scalable One-Step Exfoliation for Ionic and Molecular Nanofiltration

**DOI:** 10.1021/acsmaterialsau.5c00026

**Published:** 2025-05-14

**Authors:** Aritsa Bunpheng, Thanit Saisopa, Pawin Iamprasertkun, Anusorn Seubsai, Adisak Boonchun, Weekit Sirisaksoontorn, Wisit Hirunpinyopas

**Affiliations:** † Department of Chemistry, Faculty of Science, 426949 Kasetsart University, Chatuchak, Bangkok 10900, Thailand; ‡ Department of Applied Physics, Faculty of Sciences and Liberal Arts, 202946 Rajamangala University of Technology Isan, Nakhon Ratchasima 30000, Thailand; § School of Bio-Chemical Engineering and Technology, Sirindhorn International Institute of Technology, 37698 Thammasat University, Pathum Thani 12120, Thailand; ∥ Research Unit in Sustainable Electrochemical Intelligent, Thammasat University, Pathum Thani 12120, Thailand; ⊥ Department of Chemical Engineering, Faculty of Engineering, Kasetsart University, Bangkok 10900, Thailand; # Department of Physics, Faculty of Science, 54775 Kasetsart University, Chatuchak, Bangkok 10900, Thailand

**Keywords:** boron nitride, membrane, functionalization, sieving, filtration

## Abstract

Two-dimensional (2D) nanomaterials, such as graphene, have been widely used in various applications, such as electrodes for energy storage and laminar membranes for separations. Hexagonal boron nitride (hBN), one of the 2D materials possessing properties similar to graphene, can be used as laminar stacking laminates for separation processes due to its high filtration efficiency and solvent flow. Herein, we prepared 2D-hBN nanosheets using different nitrogen-containing precursors via facile liquid-phase exfoliation for the preparation of hBN membranes. We found that the as-prepared hBN samples exhibit unique physicochemical properties, as determined by various spectroscopic techniques, particularly near-edge X-ray absorption fine structure spectroscopy, which was used to identify the presence of defects on the hBN nanosheets. The elemental compositions of each hBN nanosheet were also revealed by an X-ray photoelectron spectroscopic technique, indicating significant changes in the B:N and B:C ratios. The hBN membranes exhibit high stability in aqueous solutions without membrane deformation. The nanochannel height of the hBN membranes was found to be *∼*0.34 nm, as determined by X-ray diffraction analysis. The membranes demonstrate excellent rejection performance for charged dye molecules (acid orange 7 and methylene blue) with high water permeation rates. This is due to electrostatic repulsion between the negatively charged surface of the hBN membranes and the charged species, as well as size exclusion from the narrow capillary channels between the stacked layered hBN nanosheets. Therefore, the hBN membranes, with their unique physicochemical properties, are promising for applications in water purification.

## Graphical abstract

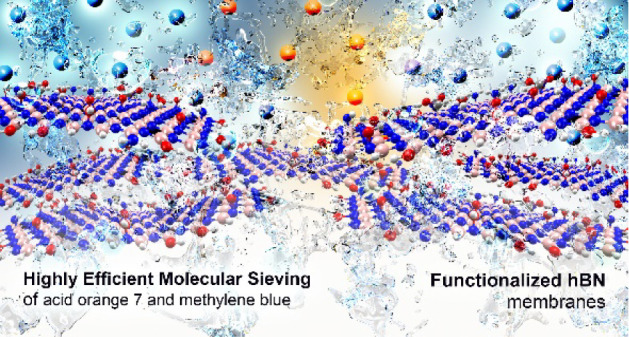

## 1 Introduction

There are many structural phases of boron nitride (BN), of which the most stable phase is hexagonal BN (hBN), possessing a structure analogue to graphite.^1,2^ The hBN nanosheet is one of the two-dimensional (2D) materials possessing physical properties close to those of graphene. Unlike carbon-based materials, BN has promising thermal strength and chemical stability under harsh conditions, which can be used in various applications requiring high mechanical strength and lightweight materials.^1–3^ Moreover, BN materials can be applied in other applications, such as high-temperature lubricants and photoluminescent devices, especially known as “superstrong” material supports in composite materials.^2^

One of the potential applications for 2D-hBN materials is membrane technology for filtration-based separation. Due to the atomic thickness and controllable nanosheet dimensions of 2D-hBN, the membrane can be formed as a laminar stacking structure that provides characteristic nanopores and nanocapillary channels for permeation properties.^1–5^ The functions of the laminar hBN membrane behave as a selective fence that can allow small molecules to transport through while rejecting larger molecules.^3–5^ Thus, the hBN dimensions (i.e., lateral length and thickness) play a significant role in the separation performance, e.g., permeability and selectivity of the solute species.^1,6^ Even though the influence of hBN dimensions is crucial for membrane performance, the surface charge of hBN nanosheets remarkably impacts on the selectivity mechanism.^1,3^ Based on the functionalization process, the surface chemistry of hBN nanosheets can be tuned to improve the permeability of the desired solutes and rejection selectivity.

A number of techniques have been successfully demonstrated to improve the surface chemistry of hBN nanosheets, including chemical modifications (urea, H_2_O_2_, etc.) to functionalize oxygen-containing functional groups on the hBN nanosheet surface^1,3,7–9^ and plasma treatment and electron beam irradiation to generate radical and oxidation reactions on the hBN nanosheets.^10,11^ Hence, the functional groups can be attached as a single bond to the unpaired B or N atom to balance the charge of the hBN nanosheets. In another way, other functional groups can be attached as a bridging bond at the opening B=N bonds (similar to the case of C=C bonds in a graphene system).^7–9^ Based on the BN synthesis process, various nitrogen-containing precursors have been widely used to synthesize BN compounds, providing characteristic physical properties such as surface area, functionality, and desired properties.^12–14^ Conversely, to the best of our knowledge, there has been no previous report on the use of different nitrogen-containing precursors during the sonication-assisted exfoliation process. We strongly believe that our studies on the influence of various nitrogen sources can potentially yield novel functionalization strategies for the structure of 2D materials.

In this work, we prepared 2D hexagonal boron nitride (2D-hBN) using various nitrogen-containing precursors through facile sonication-assisted exfoliation. The influence of nitrogen-containing precursors (i.e., nitrogen saturation, ammonia solution, and urea) on layered hBN nanosheets was investigated. The properties of hBN nanosheets (i.e., morphology, surface charge, and functionalization) were characterized using various spectroscopic techniques, such as high-resolution transmission electron microscopy, near-edge X-ray absorption fine structure spectroscopy, and X-ray photoelectron spectroscopy. The hBN nanosheets were prepared as laminar stacking membranes for ionic and molecular nanofiltration. We investigated ion filtration with different hydrated cation radii (NaCl and CaCl_2_) and charged dye molecules (acid orange 7 and methylene blue as negatively and positively charged dyes, respectively). We found that the sieving mechanisms of the hBN membranes were due to the effect of size exclusion from the narrow and tortuous nanocapillary channels inside the membranes and the electrostatic interaction between the negatively charged hBN membranes and the charged molecules. Thus, hBN membranes with unique physicochemical properties can be applied as alternative water purification-based membranes, aligning with Sustainable Development Goals (No. 6: clean water and sanitation).^15,16^

## 2 Experimental Section

### 2.1 Materials

Hexagonal boron nitride powder (∼1 μm, 98% purity), urea, and 2-propanol were purchased from Sigma-Aldrich. Polyvinylidene fluoride filters (PVDF, hydrophilic surface, 0.1 μm pore size, and 13 mm diameter) were purchased from Merck Millipore Limited. All aqueous solutions were prepared from ultrapure deionized water (Milli-Q water). Ammonia solution (28% concentration) was purchased from QRëC.

### 2.2 Preparation of Boron Nitride Dispersion and Boron Nitride Membrane

The hexagonal boron nitride (hBN) dispersion was prepared by sonication-assisted exfoliation.^5,17^ To reduce the toxicity and contamination associated with the use of organic solvents, 1 g of hBN powder was sonicated in 100 mL of a mixture of water and isopropanol (IPA) at 1:1 v/v for 24 h at 25 °C under bath sonication.^18,19^ The sonic waves were generated at a frequency of 40 kHz and a power of 300 W for the exfoliation process. The dispersion was then centrifuged twice at 3500 rpm for 30 min to remove unexfoliated materials. The exfoliated hBN dispersion was collected at the top 80% of the supernatant. This technique is referred to as centrifugation-based selection, in which the exfoliated materials (lower mass) remain dispersed in the supernatant, while the unexfoliated materials (higher mass) form a precipitate at the bottom of the centrifuge tube. The as-prepared hBN dispersion is referred to as the ex-hBN sample (schematic in Figure 1a).

**Figure 1:**
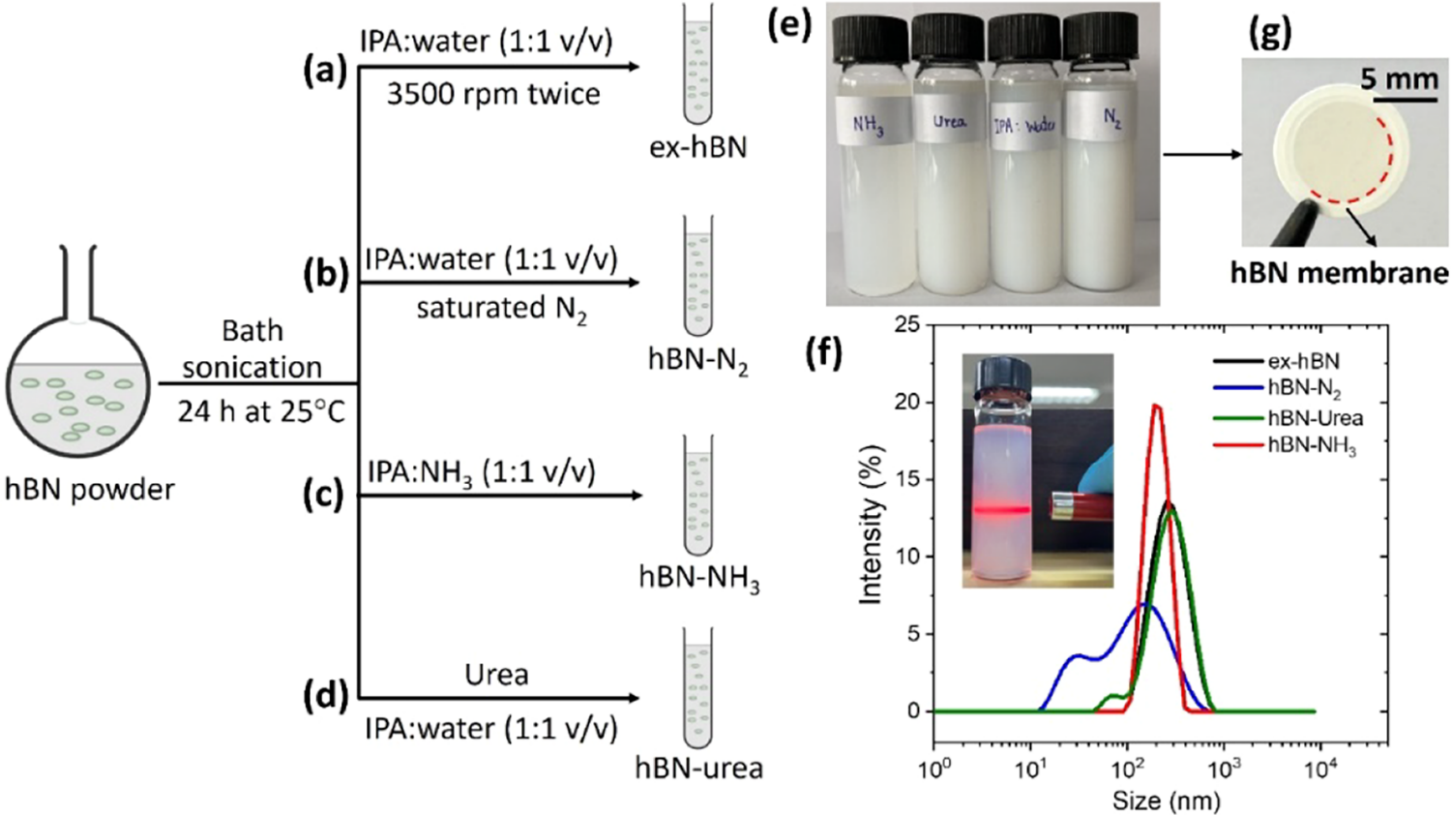
Preparation of exfoliated hBN under different nitrogen precursor sources. Schematics showing the synthesis of (a) ex-hBN, (b) hBN-N_2_, (c) hBN-NH_3_, and (d) hBN-urea dispersions. (e) Photographs of their corresponding hBN dispersions. (f) Hydrodynamic size plot of hBN nanosheets analyzed by dynamic light scattering. The inset shows the Tyndall effect of the hBN-NH_3_ dispersion. (g) Photograph of the hBN membrane on a PVDF support.

The hBN dispersions can also be prepared using different nitrogen-containing precursors, such as nitrogen saturation, ammonia solution (NH_3_), and urea, which were denoted as hBN-N_2_, hBN-NH_3_, and hBN-urea, respectively (Figure 1b–d). The exfoliation process for hBN-N_2_, hBN-NH_3_, and hBN-urea dispersions is similar to that of ex-hBN. For the preparation of the hBN-N_2_ dispersion, hBN powder was dispersed in a mixture of water and IPA (1:1 v/v) and then purged with high-purity nitrogen gas for around 30 min prior to the exfoliation process. The flask was sealed with a rubber stopper connected to a balloon of nitrogen gas to minimize nitrogen leakage. For the hBN-NH_3_ preparation, the hBN powder was sonicated in a mixture of ammonia solution and IPA (1:1 v/v) under bath sonication. For the hBN-urea preparation, 1 g of hBN powder was sonicated with 2.42 g of urea (1:1 mol ratio) in 100 mL of a mixture of water and IPA (1:1 v/v). Figure 1e shows the photograph of each hBN dispersion after the exfoliation process, exhibiting a well-dispersed solution, as determined by dynamic light scattering (DLS) in Figure 1f. Figure 1f inset shows the Tyndall effect of the exfoliated hBN dispersions when a laser was irradiated through the solution. This effect indicates the nature of colloidal nanosheets and the good stability of the exfoliated hBN dispersions. The concentration of the as-prepared hBN dispersions was determined via a weight method. Briefly, the hBN dispersion was filtered through a preweighted PVDF filter. A 5-decimal analytical balance was used to minimize the errors in measurement. The obtained hBN film on the PVDF filter was dried in an oven for 12 h and reweighted to obtain the hBN mass. This was carried out at least 3 times to get an average concentration.

The laminar hBN membrane was prepared using a programmable syringe pump at a constant rate of 10 mL h^–1^ for self-assembly stacking.^5,17^ First, the hBN dispersion was diluted 10 times in a mixture of water and IPA and then mildly sonicated for 5 min to obtain a homogeneous solution. The obtained solution was filtered through a preweighted PVDF filter and dried at 60 °C in an oven overnight. The dried membrane was weighed to determine the mass of hBN loading, (see Figure 1g). The membranes were prepared at a comparable thickness (∼10 μm) to study ionic and molecular filtration.

### 2.3 Characterization of Boron Nitride Dispersions and Membranes

The lateral length of hBN nanosheets was initially determined by the DLS technique using a Malvern Zetasizer Nano ZS90 instrument operated with a 633 nm He–Ne laser. The zeta potential of hBN nanosheets was also determined using a Malvern Zetasizer Nano ZS by using the Smoluchowski equation. The hBN dispersion for DLS and zeta potential measurements was diluted to be ∼0.01 mg mL^–1^ to minimize the nanosheet aggregation. The lateral dimension of hBN nanosheets was directly determined by transmission electron microscopy (TEM), carried out with a JEOL JEM-ARM200F instrument at an accelerating voltage of 200 eV. The sample was dropped on a copper grid (lacey carbon) and cleaned with an EC-52000IC ion cleaner to remove hydrocarbon contaminants from the specimen. The morphology and thickness of hBN membranes were determined by scanning electron microscopy (SEM) using a FEI ESEM Quanta 450 with an accelerating voltage of 15 kV. The crystal structure of hBN membranes was carried out with X-ray diffraction (XRD) with a Bruker D8 ADVANCE diffractometer with a Cu Kα source (0.154 nm). The XRD patterns were recorded over a 2θ range of 5–60° with a step size of 0.01°. The structures of hBN membranes were analyzed by Raman spectroscopy. Confocal Raman spectroscopy was performed using a Renishaw microscope with a 532 nm excitation with a power of ∼0.1%, a 50× objective lens, and a grating of 1800 l/mm to obtain a spectral resolution of ∼1 cm^–1^. Near-edge X-ray absorption fine structure (NEXAFS) measurements was performed at the Synchrotron Light Research Institute (SLRI) of Thailand with a BL3.2Ua SLRI beamline. NEXAFS measurements were carried out in partial fluorescence mode with a commercial silicon drift detector. NEXAFS spectra of the N K-edge were collected at a photon energy of 389–430 eV with an energy step of 0.1 eV. It is noted that carbon contamination on the gold mesh used for measuring photon intensity was used to calibrate the XANES spectrum. X-ray photoelectron spectroscopy (XPS) was performed with a Kratos Axis Ultra spectrometer. The Al Kα source (1486.6 eV) was used as the excitation radiation, with charge neutralization applied during measurement. All XPS spectra were calibrated with C 1s peak at a binding energy of 284.8 eV. It is important to note that the hBN specimen for XRD, Raman, NEXAFS, and XPS analyses was prepared on a PVDF filter at a comparable thickness prior to measurements.

### 2.4 Measurement of Ion Permeability

#### 2.4.1 Ion Permeability under Diffusive Pressure

At first, the hBN laminate supported on a PVDF filter was assembled between two polyethylene terephthalate (PET) sheets, bound with epoxy glue. The membrane, possessing an exposed hBN area of ∼0.26 cm^2^, was inserted into a customized H-shaped cell.^4,17^ The cell was filled with high and low solution concentrations at an equivalent volume of 25 mL as the feed (1 M) and permeate (1 mM) sides, respectively, which provided a 1000 × concentration gradient. This technique was previously reported to study ion permeability under high diffusive pressure.^4,5,17,20^ Both solutions were stirred during measurement to minimize the effect of the concentration gradient. The membrane performance was evaluated using in situ techniques for 3 h, which measured the increase in total solute species on the permeate side.^5,17^

#### 2.4.2 Ion Permeability under Forward Osmosis Pressure

The membrane performance was also determined by a forward osmosis pressure using a U-shaped cell in which the membrane was separated by two liquid sides. This technique was previously used to evaluate 2D material-based membranes such as graphene,^5,17^ graphene oxide,^20^ and MoS_2_ membranes.^4,21^ The cell was filled with an equal volume of 1 M sucrose and 0.1 M NaCl as the draw and feed sides, respectively, for 12 h at 30 °C. This technique provides an osmotic pressure gradient of ∼25 bar based on van’t Hoff’s law,^5,17,20^ which can draw water molecules from the feed side to the sucrose side. The rejection percentage (%*R*) was calculated as follows:

$$\%R=1-C_{p}/C_{f}$$

where *C*_f_ and *C*_p_ are the concentrations of charged dyes in the feed and permeate sides, respectively. The water permeance was observed with an increasing volume of the permeate side. The charged dyes (i.e., methylene blue and acid orange 7) were used as positively charged and negatively charged dyes, respectively, for the molecular sieving study. The concentration of dye on the feed side was initially set to 30 ppm, at which the change in dye concentrations was determined by a UV–vis spectrophotometer.

## 3 Results and Discussion

### 3.1 Boron Nitride Dispersion and Boron Nitride-Based Membranes

The average concentrations of ex-hBN, hBN-N_2_, hBN-NH_3_, and hBN-urea were 0.98 mg mL^–1^, 2.27 mg mL^–1^, 0.26 mg mL^–1^, and 0.88 mg mL^–1^, respectively (Figure 1e). Figure 1f shows the size distribution of each hBN nanosheet, as analyzed by the DLS technique. The average hBN sizes of each hBN nanosheet were in a similar range of 100–300 nm. The charge on each hBN nanosheet was measured using the zeta potential, which corresponds to −17.4, −20.3, −23.8, and −20.6 mV for ex-hBN, hBN-N_2_, hBN-NH_3_, and hBN-urea, respectively. The results showed that the modified hBN nanosheets have a more negatively charged surface than that of ex-hBN, suggesting chemical functionalization on the hBN surface after exfoliation (discussed in the NEXAFS and XPS sections). All hBN dispersions exhibited homogeneity for several weeks due to the electrostatic repulsion of negatively charged hBN nanosheets. The stability of colloidal hBN was shown at different time intervals, as shown in Figure S1. It is clearly seen that a Tyndall effect of hBN dispersions can still be demonstrated after 2 weeks, indicating high stability with no observed sedimentation of the hBN. In addition, the dimensions (lateral size and shape) of each hBN sample were directly measured by transmission electron microscopy (TEM) images, as shown in Figure 2. The shape of each hBN nanosheet was observed as a nanosheet-like morphology with a smooth nanosheet surface, unlike the one under ammonia conditions. This might result from the effect of hydroxide functionalization leading to a large amount of defect formation.^9^ Figure 2a–d shows the lateral size histograms of ex-hBN, hBN-N_2_, hBN-NH_3_, and hBN-urea, respectively. The lateral nanosheet length was measured along the major length (see the solid line), which was counted as at least 150 nanosheets for each individual hBN nanosheet. The mean lateral nanosheet size for ex-hBN, hBN-N_2_, hBN-NH_3_, and hBN-urea were 146 ± 39 nm, 106 ± 29 nm, 118 ± 21 nm, 112 ± 22 nm, respectively. The low- and high-magnification TEM images of each hBN sample are also shown in Figure S2. It is clearly seen that the lateral sizes observed directly from TEM analysis are slightly smaller than those from DLS analysis, resulting from the solvation effect on hBN nanosheets.

**Figure 2:**
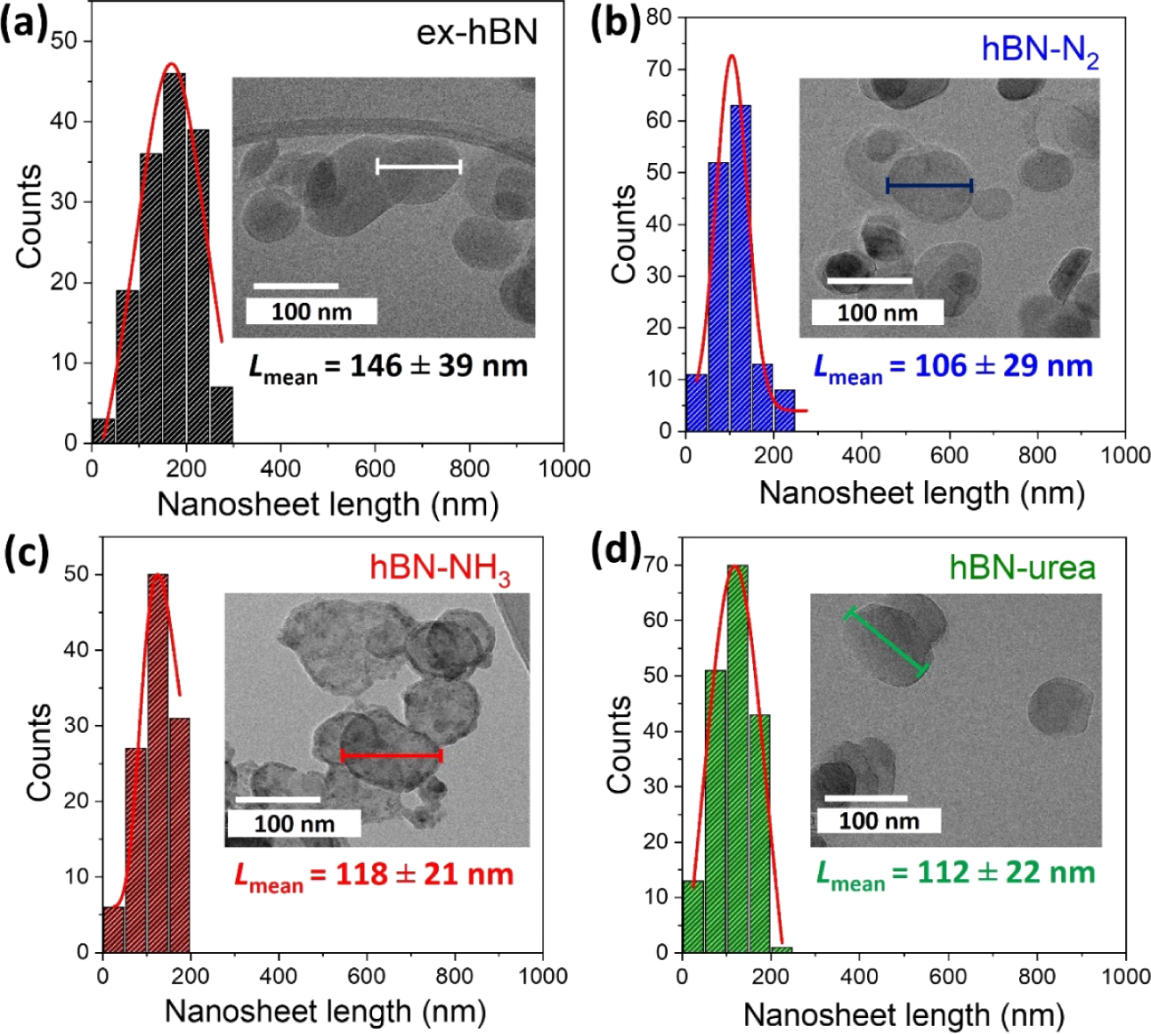
Histogram showing the lateral size of (a) ex-hBN, (b) hBN-N_2_, (c) hBN-NH_3_, and (d) hBN-urea, as measured by statistical TEM. The histograms were fitted with the Lorentzian function, as shown in the red solid line. The insets show their corresponding bright-field TEM images.

In order to further characterize the morphology of exfoliated hBN nanosheets, Figure 3 shows typical and high-resolution TEM images of the hBN-N_2_ sample prepared in IPA:water with nitrogen saturation. It was found that the morphology of hBN-N_2_ was similar to that of coin-like nanosheets. The exfoliated hBN nanosheets had ultrathin and transparent structures with a few hBN layers (Figure 3a,b), whereas bulk hBN powder with large chunk particles was very thick and opaque (see Figure S3). It is clearly evident that the hBN nanosheets were exfoliated into very thin nanosheet layers, leading to a decrease in the number of hBN layers. Also, Figure 3c shows the HR-TEM images of the hBN-N_2_ nanosheets. The symmetry stacking orders of hBN structures were found to be AA- and AB-stacked regions (Figure 3c inset), attributed to Bernal-stacked hBN.^22,23^ The exfoliated hBN prepared by sonication-assisted exfoliation possesses few-layer nanosheets, which is in agreement with previously reported works.^24–26^ In addition, the selected area electron diffraction (SAED) pattern of the hBN-N_2_ nanosheets was observed as the hexagonally symmetric atomic configuration of the hBN structure, which exhibited six-fold symmetrical spots, as shown in Figure 3d.^24,25^ These results suggest that the crystal structure of exfoliated hBN nanosheets was still retained during the sonication process.

**Figure 3:**
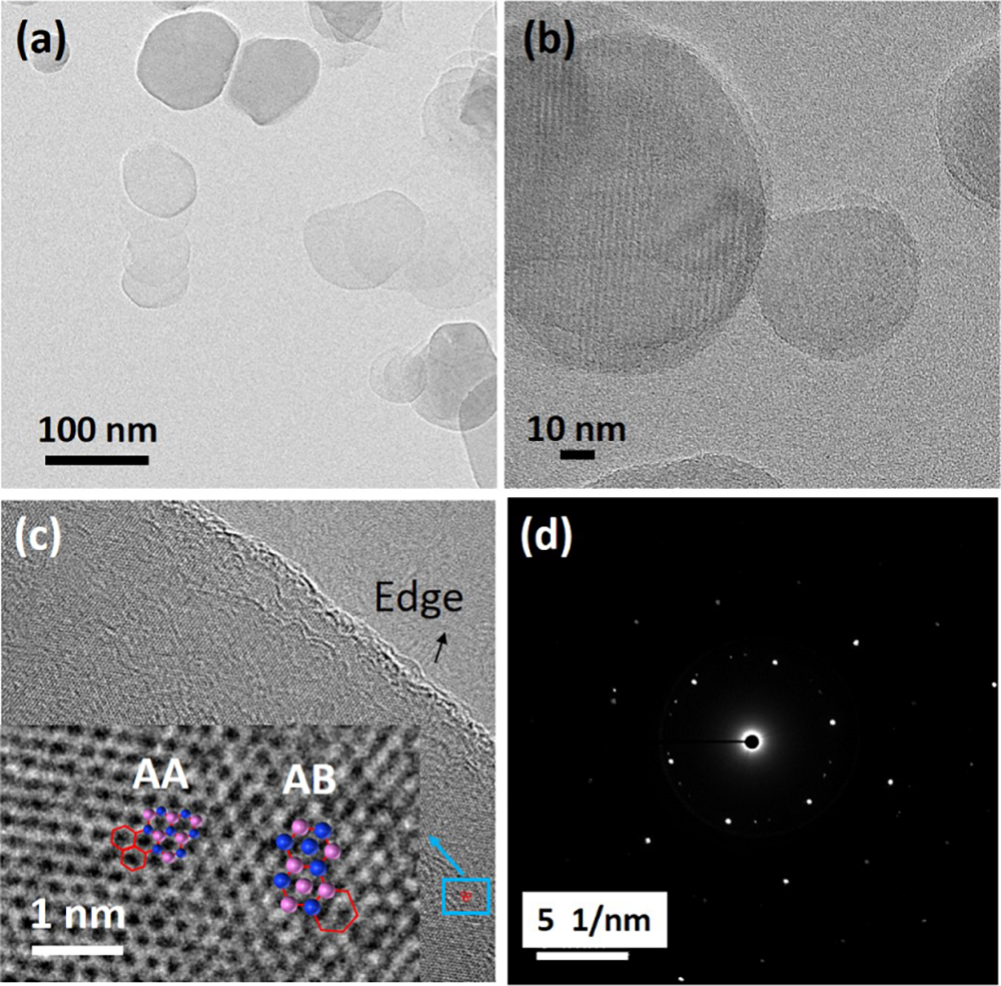
TEM characterization of exfoliated hBN-N_2_ nanosheets. (a) Low- and (b) high-magnification TEM characterizations showing few-layer exfoliated hBN nanosheets. (c) High-resolution TEM image showing the edge of a few-layer hBN nanosheet as well as a representative crystal structure of the basal plane of hBN showing the AA and AB stacking orders. Note that boron and nitrogen atoms are pink and blue spheres, respectively. (d) The corresponding selected area electron diffraction (SAED) pattern of hBN-N_2_ nanosheets.

Figure 4a–d shows top-down SEM images of ex-hBN, hBN-N_2_, hBN-NH_3_, and hBN-urea, respectively. The membranes exhibited disordered patterns of nanosheets forming stacked laminar hBN structures. The lateral size of each hBN nanosheet agrees well with the size measured by TEM and DLS analyses. Moreover, cross-sectional SEM images of hBN membranes are also shown in Figure 4e–h with their enlarged SEM images. It was found that the membranes possess a well-defined laminar hBN structure, which was formed by the individual hBN nanosheets on a PVDF filter. The membranes have a closely packed structure of restacked hBN nanosheets, which provide massive nanocapillary channels inside the membranes. This offers not only long capillary nanochannels but also generates tortuous pathway for ionic and molecular sieving studies. The hBN membranes were prepared at a comparable thickness (10 μm thick) for all filtration measurements, as determined by the thickness calibration in Figure S4. Figure S5 shows the elemental mapping (boron, nitrogen, carbon, and fluorine elements) of the cross-sectional hBN membrane, indicating that hBN nanosheets mostly assemble on a PVDF filter with a small fraction of nanosheets embedded in a porous PVDF filter.

**Figure 4:**
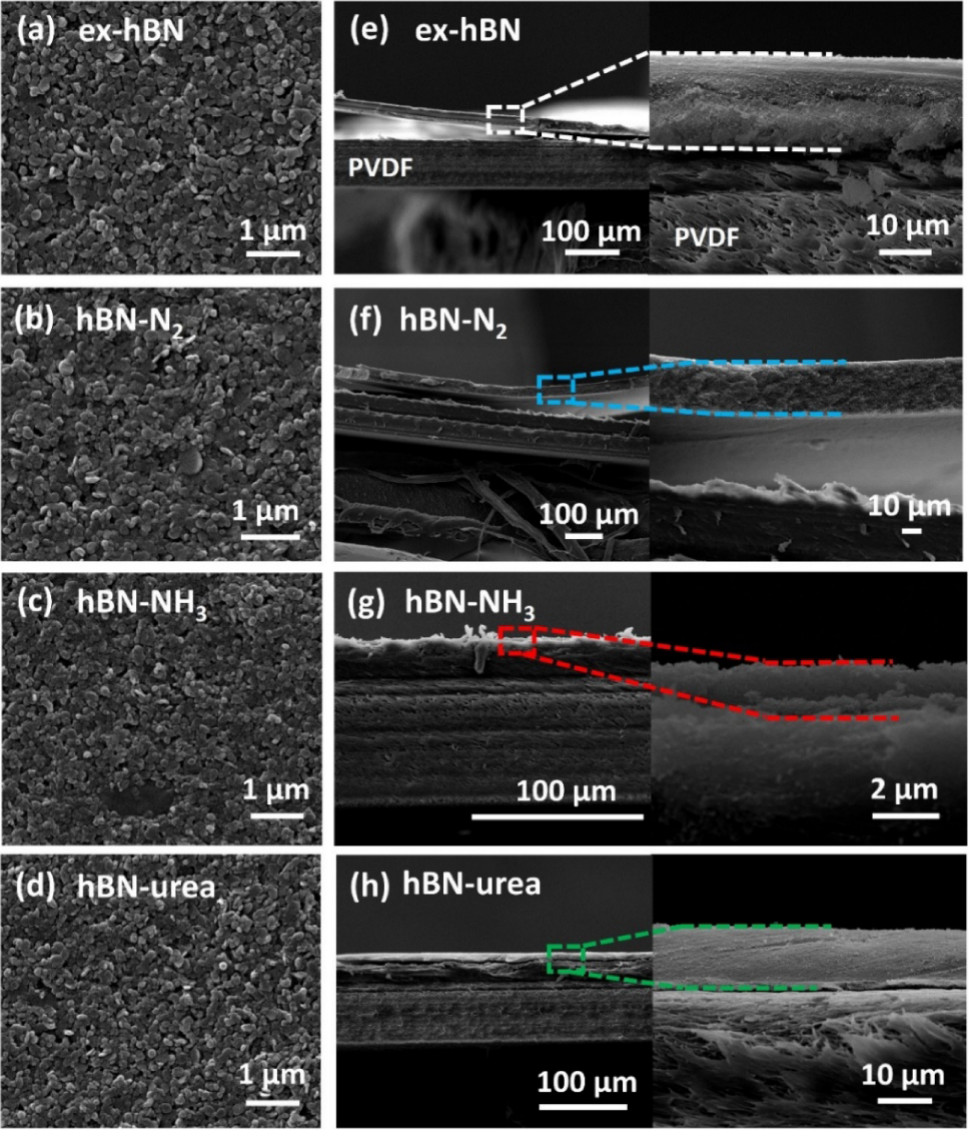
Morphologies of the hBN membranes. Top-down SEM images of (a) ex-hBN, (b) hBN-N_2_, (c) hBN-NH_3_, and (d) hBN-urea. (e–h) Cross-sectional SEM images of their corresponding laminar hBN membrane with their enlarged SEM images.

To understand the laminar packing structure of hBN membranes, the powder X-ray diffraction (PXRD) patterns containing the (002) peak of hBN and a peak of the PVDF filter are shown in Figure 5a. The wide PXRD patterns of each membrane are also shown in Figure S6a. Note that all PXRD patterns were calibrated using a reference PVDF peak at 2θ of 20.17°.^4^ It was found that the (002) peak of all hBN membranes is broader (with a wider FWHM) when compared to that of a bulk material (see Figure S6b). This is due to the low crystalline structure of hBN nanosheets during the sonication process, resulting in a decrease in lateral nanosheet size and the number of layers. This agrees well with exfoliated 2D materials prepared by the sonication-assisted exfoliation process.^4,5,17,20^

**Figure 5:**
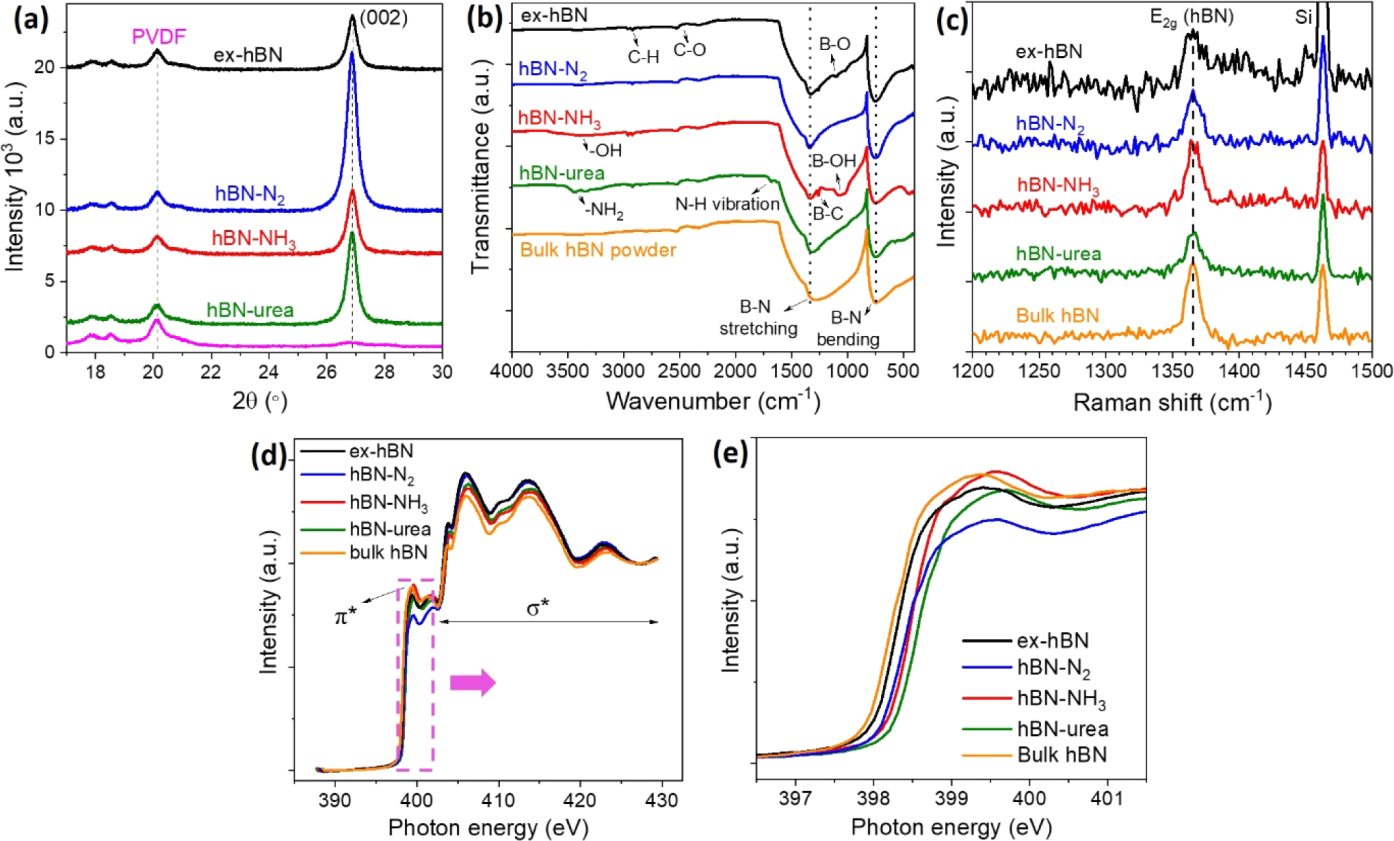
(a) PXRD pattern of hBN membranes. (b) Comparing Fourier transform infrared (FT-IR) spectra of each hBN membrane. (c) Raman spectra of various hBN membranes compared to the starting hBN powder. (d) Nitrogen *K*-edge spectra of each hBN sample determined by near-edge X-ray absorption fine structure spectroscopy with (e) their enlarged section showing a shift of X-ray absorption. Note that all nitrogen *K*-edge spectra were calibrated with a C 1s peak.

To analyze the chemical functionalization of hBN samples after the exfoliation process, the Fourier transform infrared (FTIR) technique was used to determine the bond stretching between the nitrogen and boron atoms (lattice vibration modes). Figure 5b shows the FTIR spectrum of exfoliated hBN samples compared with bulk hBN powder (the starting material). The hBN samples exhibit strong absorption at ∼1300 cm^–1^ and ∼750 cm^–1^ corresponding to B–N stretching (the E_1u_ mode, in-plane ring vibration) and B–N bending (the A_2u_ mode, out-of-plane vibration), respectively, which agree well with previously reported spectra.^1,27^ The FTIR spectra of individual hBN samples are shown in Figure S7, where all absorption peaks can be clearly observed. It was clearly seen that broad peaks at ∼3300 cm^–1^ and ∼1070 cm^–1^ for the hBN-NH_3_ are attributed to −OH and B–OH vibrations.^28^ These peaks indicate hydroxyl functionalization for hBN-NH_3_ when the ammonia solution was used in the exfoliation process. A tiny absorption peak at ∼1100 cm^–1^ was identified in the ex-hBN spectrum, which was assigned to the B–O deformation. This may originate from oxidative reactions on the hBN surface.^27,29^ These peaks (both B–OH and B–O vibrations) were not found in hBN-N_2_ and hBN-urea as compared to the starting hBN powder. However, it should be noted that there are additional absorption peaks at approximately 3300–3450 cm^–1^ for the hBN-urea spectrum, which correspond to N–H stretching vibrations.^1,30,31^ The appearance of these peaks can confirm the functionalization of the amino group, which occurred at the defects and edges of exfoliated BN nanosheets.^1,8,30^ The structures of exfoliated hBN materials were also analyzed by Raman spectroscopy, as shown in Figure 5c. The Raman spectra show that a band centered at ∼1366 cm^–1^ corresponds to the in-plane E_2g_ vibrational mode of hBN.^1,7,24^ It was seen that the FWHM of hBN membranes (∼15.7 cm^–1^) are wider than that of a starting material (∼10.8 cm^–1^), indicating the presence of few layers of exfoliated hBN nanosheets (as discussed in TEM images of Figure 3). This is due to a weaker interaction between the few hBN layers after the exfoliation process.^1,32^

Moreover, we also determined each hBN sample using near-edge X-ray absorption fine structure (NEXAFS) spectroscopy, as shown in Figure 5d,e. NEXAFS spectroscopy is an effective tool for determining the physical and chemical properties of material surfaces, particularly for identifying the local bonding environment.^33–35^ This technique can be used to identify the phases of BN structures as well as defect formations and the crystallinity of BN samples.^34,35^ Figure 5d shows the nitrogen *K*-edge spectra of hBN samples, demonstrating the electronic transition of 1s electrons into the π* (∼399.4 eV) and σ* (high-energy features: 403.8, 406.0, 413.8, and 423.2 eV) antibonding orbital states of the sp^2^ hBN.^34,35^ These spectra reveal that the B–N bonds are sp^2^-hybridized, implying the hexagonal structure of exfoliated hBN samples, unlike the one for cubic hBN structure (sp^3^ hybridization).^35–37^ Also, the π*/σ* intensity ratio in exfoliated hBN samples is similar to that of bulk hBN powder, confirming the sp^2^-BN-like configuration.^38^ This also corresponds to the SAED and XRD analyses (Figures 3d and 5a). In addition, the presence of defects on exfoliated hBN was also determined by the NEXAFS spectra. It is worth noting that the N *K*-edge spectra of exfoliated hBN samples with different nitrogen-containing precursors were slightly shifted, as shown in the enlarged spectra in Figure 5e. The shift of energy might result from the existence of defect sites (e.g., nitrogen vacancies, oxidative reactions) as well as chemical functionalization (i.e., −OH and −NH_2_ functional groups) on the surface of hBN nanosheets.^35–37,39^ Further chemical compositions are discussed in the XPS section.

To get more supportive data, X-ray photoelectron spectroscopy (XPS) was also used to further analyze the chemical compositions of the exfoliated hBN sample, as shown in Figure 6. Figure 6a shows the survey-scan XPS spectra of various exfoliated hBN samples and bulk hBN powder, exhibiting the presence of B 1s, N 1s, C 1s, and O 1s. The atomic percentages of each element are shown in Table S1. Figure 6b shows the B:N (left *y*-axis) and B:C (right *y*-axis) atomic ratios for all hBN samples. The B:N atomic ratios are 1.28 (ex-hBN), 1.24 (hBN-N_2_), 1.23 (hBN-NH_3_), 1.17 (hBN-urea), and 1.15 (bulk hBN powder). It was found that ex-hBN has a higher B:N atomic ratio that is attributed to the absence of nitrogen atoms (nitrogen vacancies) on the hBN structure during the exfoliation process (see Table S1). In other words, it should have an additional bonding state on the structure, e.g., the BN_*x*_O_*y*_ component, referring to the formation of defect sites of the exfoliated hBN (further discussed in Figure 6c,d).^37^ On the other hand, using nitrogen-containing precursors can effectively minimize the vacancies, particularly in hBN-urea, which possesses a ratio close to the starting material (bulk hBN). Moreover, the carbon content was also determined using the B:C atomic ratio (right *y*-axis in Figure 6b). The B:C atomic ratios of exfoliated hBN samples are far lower than the bulk one, which has a significant change after the exfoliation process, indicating a considerable amount of carbon content. The increased carbon content mainly originated from 2-propanol used during the exfoliation process. However, it is worth noting that the addition of N_2_, NH_3_, and urea can significantly reduce carbon-containing functionality from the hBN surface when compared with ex-hBN. Thus, the use of nitrogen-containing precursors during liquid-phase exfoliation can not only retain the chemical composition but also reduce the carbon content on the hBN structure.

**Figure 6:**
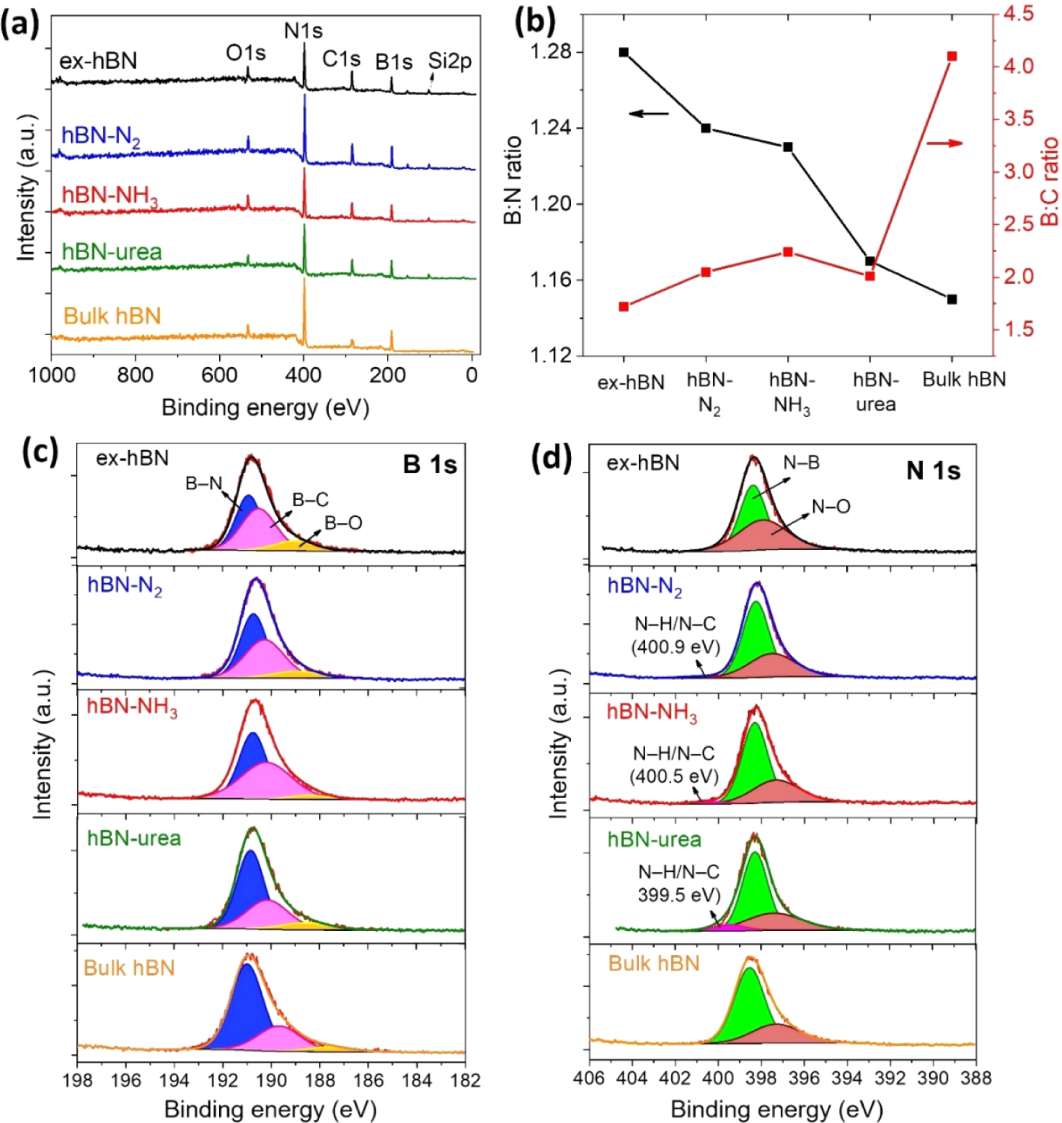
XPS analysis of each hBN sample. (a) Survey scan XPS spectra of the exfoliated hBN sample compared with bulk hBN powder. (b) B:N (left *y*-axis) and B:O (right *y*-axis) atomic ratios of different hBN samples. High-resolution XPS spectra of (c) B 1s and (d) N 1s peaks with their fitting curves of each exfoliated hBN samples and bulk hBN powder.

The high-resolution XPS spectra of B 1s and N 1s are shown in Figure 6c,d, respectively. As shown in Figure 6c, the B 1s peak was deconvoluted into three main peaks associated with B–N, B–C, and B–O bonds. The first peak (blue area at ∼190.7–191.0 eV) is the B–N bond, which is from the hBN-like structure (sp^2^ hybridized).^36,37,40^ The second peak (pink area at ∼189.7–190.3 eV) is the B–C bond, which may arise from BC or BCN compounds.^38,40^ The shoulder peak at ∼188.7–189.9 eV is ascribed to B–O bonds, which originate from the starting material (bulk hBN) as well as the exposure of the hBN film to ambient conditions.^1,30^ In addition, we also found that the peak area of the B–N bond of each sample increased when nitrogen-containing precursors were added, especially for hBN-urea (similar to the bulk sample). Figure 6d shows the N 1s peak of all samples, which were deconvoluted into two main peaks. The major component appearing at ∼398.4 eV (green area) originates from the N–B bond, while the minor component appearing at ∼397.5 eV can be assigned to the presence of N–O bonds.^38,40^ Indeed, the hBN samples prepared using N_2_, NH_3_, and urea exhibit an additional signal of approximately 399.5–400.9 eV, which was assigned to N–H and N–C bonds.^30,31,40^ This is attributed to the chemical functionalization (e.g., -NH_2_) at the defect sites and edges of hBN nanosheets. The high-resolution XPS spectra of C 1s and the peaks of O 1s are shown in Figure S8 with their deconvoluted components.

#### 3.1.1 Ionic and Molecular Sieving Performance

##### 3.1.1.1 Ionic Sieving under Diffusive Pressure

Figure 7 shows ionic and molecular sieving in the hBN membranes. The cross section of the hBN membrane is shown in Figure 7a with the corresponding schematic in Figure 7b, demonstrating the laminar structure of stacked hBN nanosheets for ionic and molecular sieving. The channel height of hBN membranes can be estimated to be ∼0.34 nm, as determined using *d*-spacing of the XRD analysis (Figure 5a).

**Figure 7:**
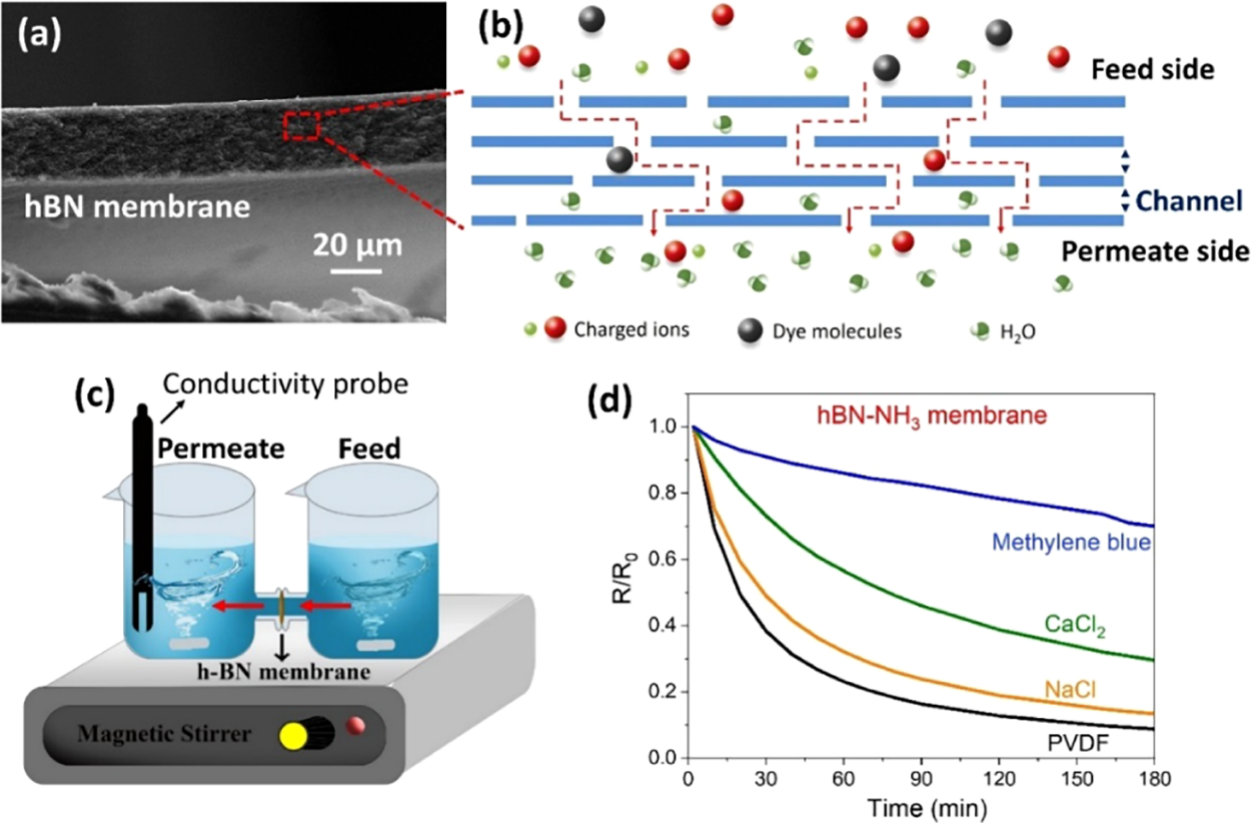
Ionic and molecular transport through the hBN membrane. (a) Cross-sectional SEM image of the laminar hBN membrane with (b) schematic showing ionic and molecular transport through nanocapillary channels inside the membrane. (c) Schematic showing the experimental apparatus for ion and molecular permeability at which the membrane was inserted between two reservoirs containing high (feed side) and low concentration (permeate side). (d) A plot comparing the relative resistivity (*R*/*R*_0_) of the permeate side of the hBN-NH_3_ membrane. Note that the concentration of the permeate side was 1000 times lower than that of the feed side.

To test the permeation of ionic and molecular solutes, the hBN membranes were initially evaluated with various solutes, including NaCl, CaCl_2_, and methylene blue, using a customized H-shaped cell, as shown schematically in Figure 7c. The cell was filled with high (1 M; feed side) and low (1 mM; permeate side) solution concentrations at an equivalent volume (25 mL) to minimize hydrostatic pressure. This technique can provide a high concentration gradient (diffusive pressure) across the hBN membrane. The property of ionic and molecular sieving was measured using the in situ conductivity technique, in which the change in ionic concentration on the permeate side was monitored for over 3 h. Figure 7d illustrates the relative resistivity of various solute species, i.e., hydrated cation diameters, such as Na^+^ (*D*_H_ = 7.16 Å), Ca^2+^ (*D*_H_ = 8.24 Å), as well as charged molecules such as methylene blue (dimensions = 3.3 × 7.6 × 17.0 Å), for a 10 μm thick hBN-NH_3_ membrane.^41,42^ The plots compare the ionic permeability of NaCl, CaCl_2_, and methylene blue compared to a bare PVDF filter. This can allow us to determine the change in total ionic solutes (positively charged ions and Cl^–^) on the permeate side of the membranes. It was found that the relative resistivity for methylene blue had slightly dropped, while the smaller cations (Na^+^ and Ca^2+^) changed dramatically after 3 h. This result indicates that methylene blue, possessing a larger molecule, can transport through the membrane more slowly than the small ionic solutes, which is due to the effect of the size exclusion mechanism.

##### 3.1.1.2 Ionic Sieving under Forward Osmosis Pressure

To evaluate the rejection properties and water permeability, we also tested the performance of hBN membranes using a forward osmosis technique. Figure 8a shows the experimental apparatus (a U-shaped cell), in which the cell was filled with equal volumes of 30 ppm dye solution and 1 M sucrose solution as the feed and draw sides of the hBN membrane, respectively, for 12 h. Note that the hBN side faced the dye solution for all experiments. The concentration gradient between both sides can generate osmotic pressure of up to ∼25 bar (van’t Hoff’s law),^43^ which can pull water molecules from the feed to the sucrose side of the membrane.^5,17,44^ The water permeability was measured by an increase in the volume of the draw side, as reported in the unit of L m^–2^ h^–1^ bar^–1^. The dye rejection was determined as *R*_dye_% = 100­(1 – *C*_d_/*C*_f_), where *C*_d_ and *C*_f_ are the dye concentrations in the draw and feed sides, respectively, as measured by UV–vis spectrophotometry. This technique has been widely used to evaluate the performance of laminar 2D membranes such as graphene, GO/rGO, and MoS_2_ membranes.,^4,5,17,20,21^

**Figure 8:**
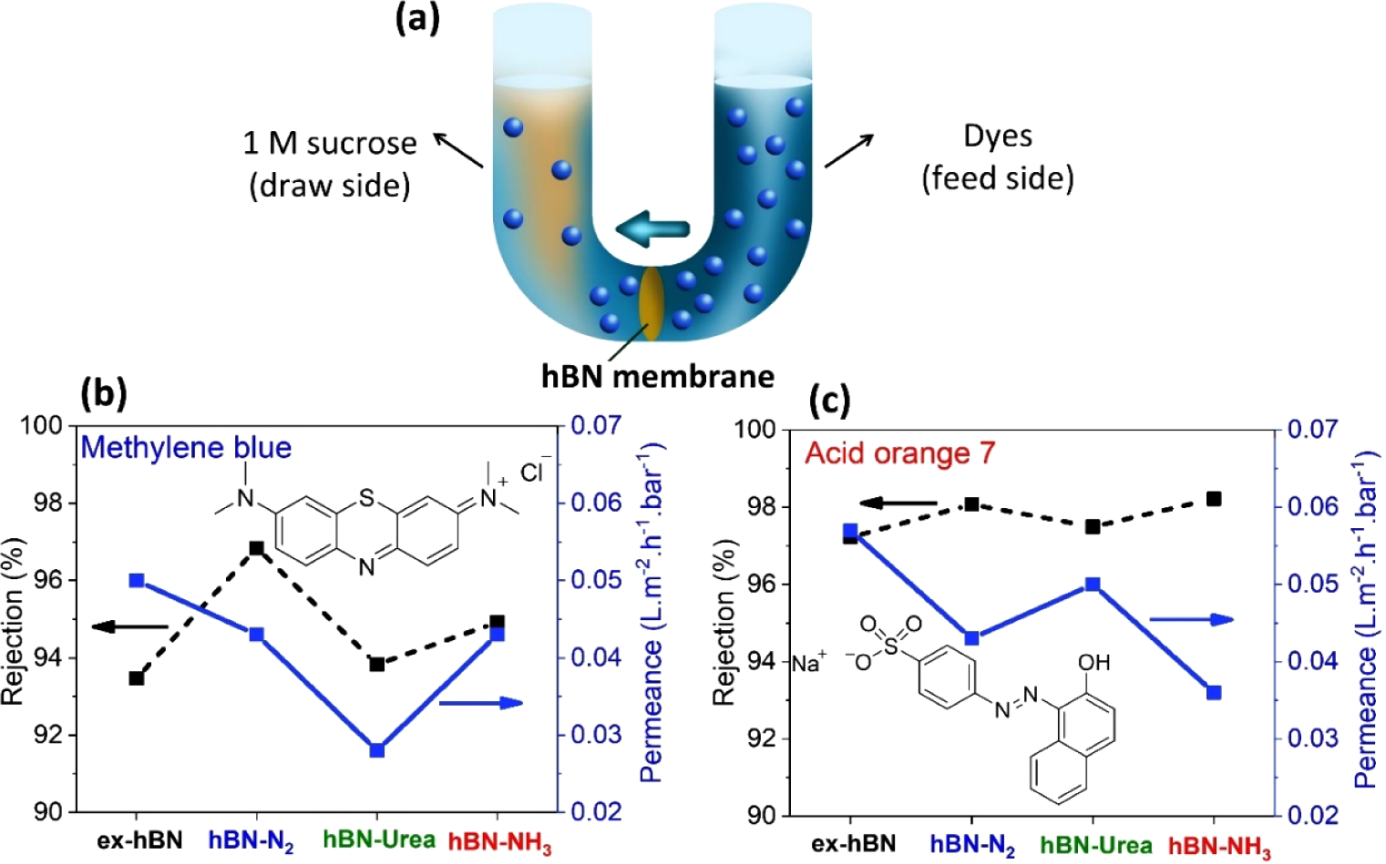
Dye rejection and water permeation through hBN membranes under forward osmotic pressure. (a) Schematic of the experimental apparatus containing permeant species and sucrose as feed side and draw side, respectively. Plots comparing (b) methylene blue rejection and (c) acid orange 7 rejection percentage on the left *y*-axis and their corresponding water permeation rate on the right *y*-axis under osmotic pressure (25 bar) using each hBN membrane at comparable thickness (10 μmthick). The rejection percentage was calculated using *R*_dye_% = 1 – C_d_/C_f_, as measured by UV–vis spectrophotometry for the dye concentration.

Figure 8b,c illustrates the dye rejection (a black line on the left *y*-axis) and water permeance (a blue line on the right *y*-axis) for as-prepared hBN membranes. For a positively charged dye molecule, the rejections of methylene blue (*R*_MB_%) are around 94–97%, with a water permeance of 0.03–0.05 L m^–2^ h^–1^ bar^–1^ for all hBN membranes, as shown in Figure 8b. In addition, in Figure 8c, for negatively charged dye molecules, the rejections of acid orange 7 (*R*_AO7_%) are about 97–98% with a water permeance of 0.04–0.06 L m^–2^ h^–1^ bar^–1^ for the as-prepared hBN membranes. It is worth noting that the AO7 rejection of hBN membranes is significantly higher than that of the MB rejection. This is due to the effect of electrostatic repulsion between negatively charged hBN membranes (average ζ-potential = ∼−20 mV) and negatively charged AO7 dye molecules. The dimensional sizes of MB and AO7 molecules are shown in Table S2. Interestingly, the water permeabilities of functionalized hBN membranes (hBN-N_2_, hBN-urea, and hBN-NH_3_) for both MB and AO7 are slightly lower than that of ex-hBN, see Figure 8b,c. This observation can be explained by the fact that the change in physicochemical properties can induce defect formation on the hBN surface (discussed in FT-IR and NEXAFS in Figure 5), which can enhance the degree of surface roughness. This leads to higher hydraulic friction of the rough and functionalized surface, resulting in the retardation of water permeance. This finding is consistent with water transport through functionalized graphene (GO/rGO) and functionalized MoS_2_ membranes.^17,42,45^ In addition, the rejection efficiency and water permeance of the as-prepared hBN were compared with previously reported laminar stacked membranes (see Table S3).

## 4 Conclusions

In summary, we demonstrated a facile functionalization technique on hBN nanosheets under various nitrogen-containing precursors via one-step exfoliation. The membranes were prepared by filtering an exfoliated hBN dispersion through a PVDF filter with an adjustable thickness. The membrane, with unique physicochemical properties, exhibited excellent molecular rejection and high water permeability. This is due to the effects of size exclusion between the channel height inside the membrane and solute species, as well as the electrostatic interaction between charged hBN membranes and charged dye molecules. Moreover, the hBN membranes showed high stability in an aqueous solution, with no observed swelling, unlike GO/rGO and MXene systems. Thus, the tunability of hBN membranes with a facile exfoliation technique can be an excellent candidate for water purification technologies.

## Supplementary Material



## Notes

Further sample characterizations, including TEM, SEM, EDX, PXRD, FTIR, XPS, thickness calibration of the membrane, and comparison of literature value of membrane performance (PDF)
