# Supplementary material for: Tuning Physicochemical Properties of Boron Nitride-Based Membranes via Scalable One-Step Exfoliation for Ionic and Molecular Nanofiltration
Source: ACS Mater Au. 2025 May 14;5(4):687–97. doi: 10.1021/acsmaterialsau.5c00026 (PMC12257375; doi:10.1021/acsmaterialsau.5c00026)
Supplement: Supplementary file 1 [file mg5c00026_si_001.pdf]

Supporting Information

Tuning Physicochemical Properties on Boron Nitride-based Membranes via Scalable One-Step Exfoliation for Ionic and Molecular Nanofiltration

Aritsa Bunpheng [a, c], Thanit Saisopa [b], Pawin Iamprasertkun [c, d], Anusorn Seubsai [e], Adisak Boonchun [f], Weekit Sirisaksoontorn [a], Wisit Hirunpinyopas [a*]

[a] *Department of Chemistry, Faculty of Science, Kasetsart University, Chatuchak, Bangkok, 10900, Thailand*
[b] *Department of Applied Physics, Faculty of Sciences and Liberal Arts, Rajamangala University of Technology Isan, Nakhon Ratchasima, 30000, Thailand*
[c] *School of Bio-Chemical Engineering and Technology, Sirindhorn International Institute of Technology, Thammasat University, Pathum Thani, Thailand 12120*
[d] *Research Unit in Sustainable Electrochemical Intelligent, Thammasat University, Pathum Thani, Thailand 12120*
[e] *Department of Chemical Engineering, Faculty of Engineering, Kasetsart University, Bangkok 10900, Thailand*
[f] *Department of Physics, Faculty of Science, Kasetsart University, Chatuchak, Bangkok, 10900, Thailand*

Keywords: boron nitride, membrane, sieving, filtration

***Corresponding author;** e-mail: wisit.hi@ku.ac.th (W. Hirunpinyopas)

Contents:

1. The stability of hBN dispersions
2. TEM images of exfoliated hBN
3. SEM images of bulk hBN powder
4. The thickness calibration of hBN membranes
5. The elemental mapping of hBN membrane
6. PXRD patterns of hBN membranes
7. FTIR spectra of hBN samples
8. XPS analysis of hBN samples
9. Comparison of literature value of 2D materials-based membranes for dye removal
10. Supporting references

1. The stability of hBN dispersions

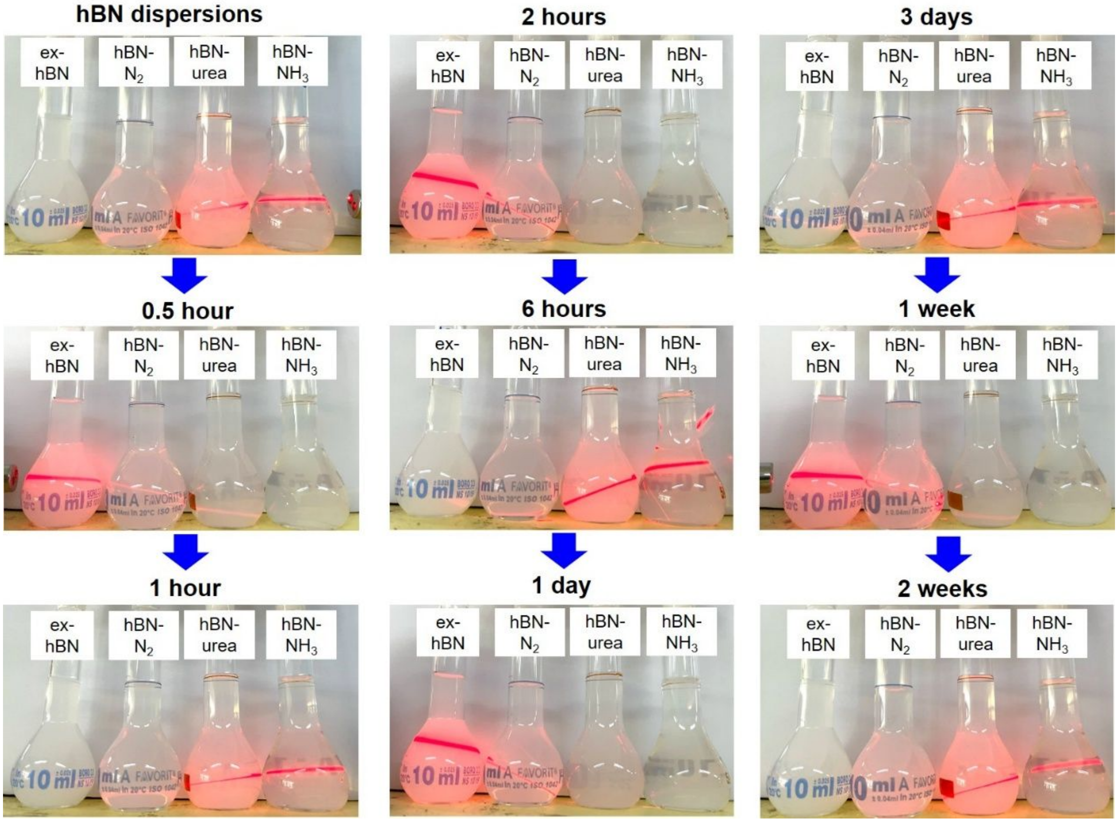

Figure S1. The stability of hBN dispersions at different time intervals; 0.5 h, 1 h, 2 h, 6 h, 1 day, 3 days, 1 week, and 2 weeks. A Tyndall effect of hBN dispersions was also demonstrated, when a laser was irradiated through the solution. Note the hBN dispersion was prepared at a comparable concentration (~0.1 mg mL^{-1}).

2. TEM images of exfoliated hBN

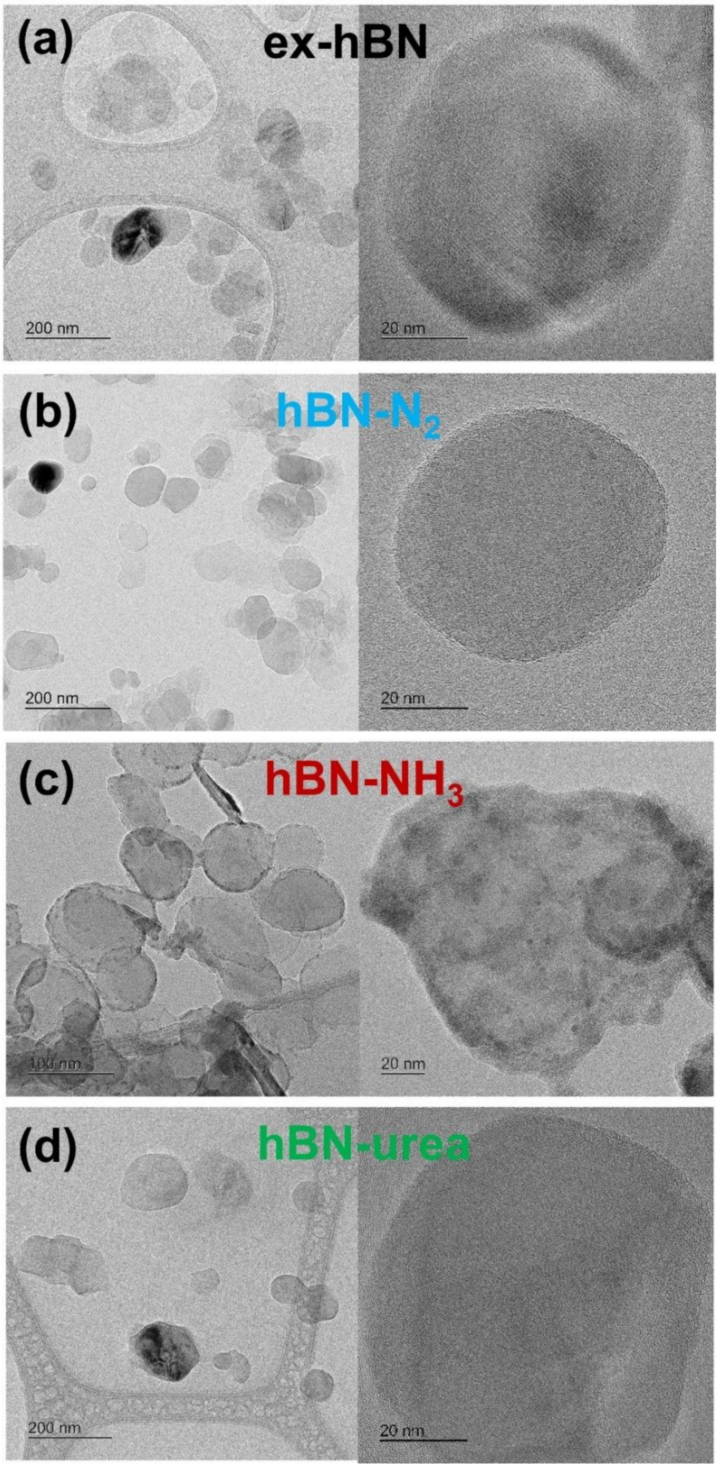

Figure S2. TEM images of (a) ex-hBN, (b) hBN-N_2, (c) hBN-NH_3, and (d) hBN-urea showing low and high-magnifications.

3. SEM images of bulk hBN powder

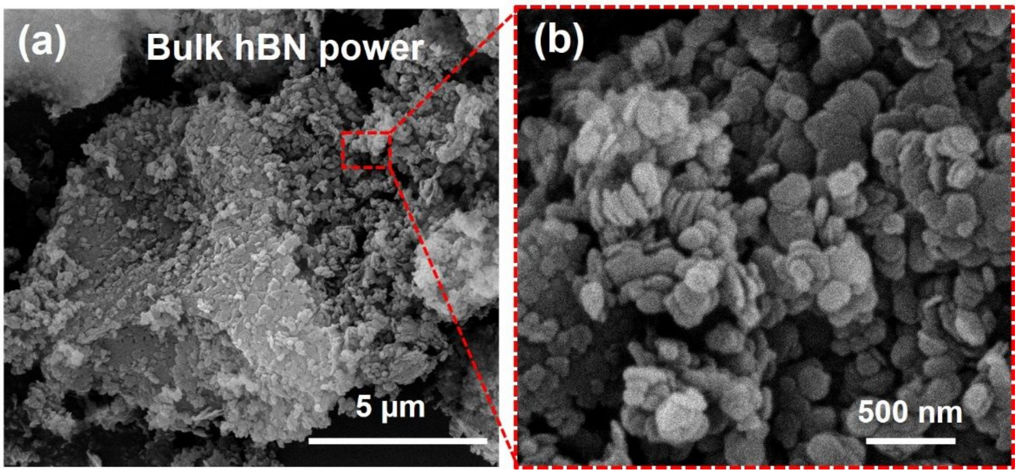

Figure S3. SEM images bulk boron nitride power (a) low and (b) high magnifications showing the large particle of boron nitride power before exfoliation process.

4. The thickness calibration of hBN membranes

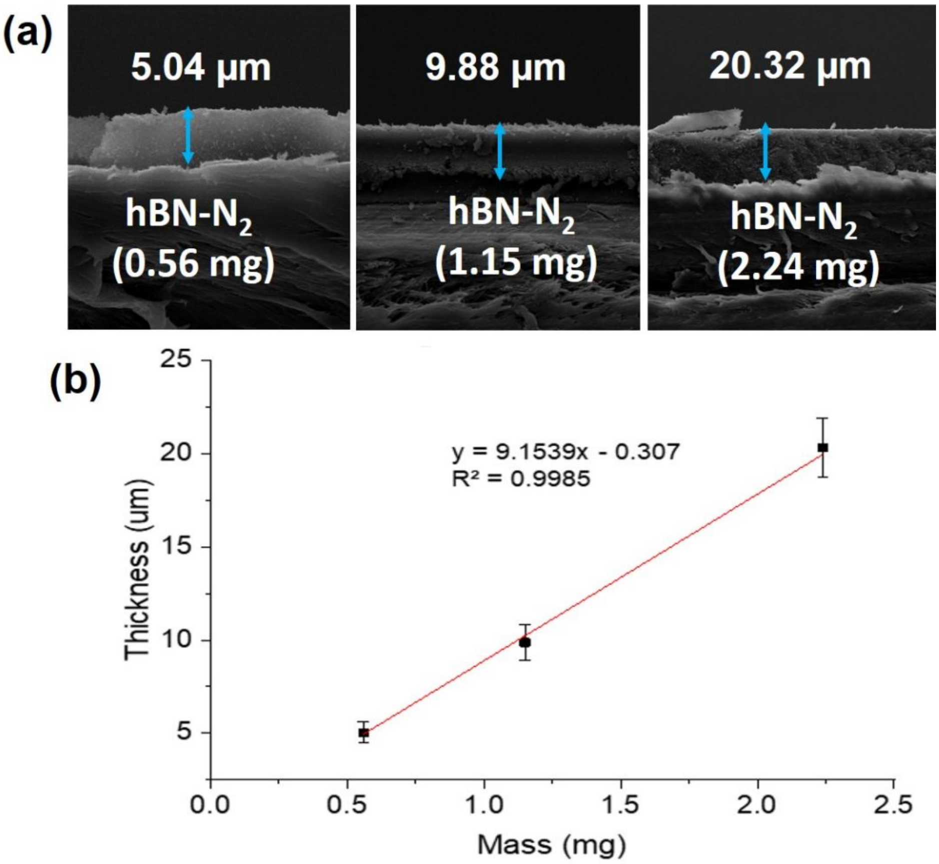

Figure S4. (a) Cross-sectional SEM images showing the hBN thickness at different hBN loading on a PVDF filter. (b) Thickness calibration of hBN membrane as a function of hBN mass loading.

5. The elemental mapping of hBN membrane

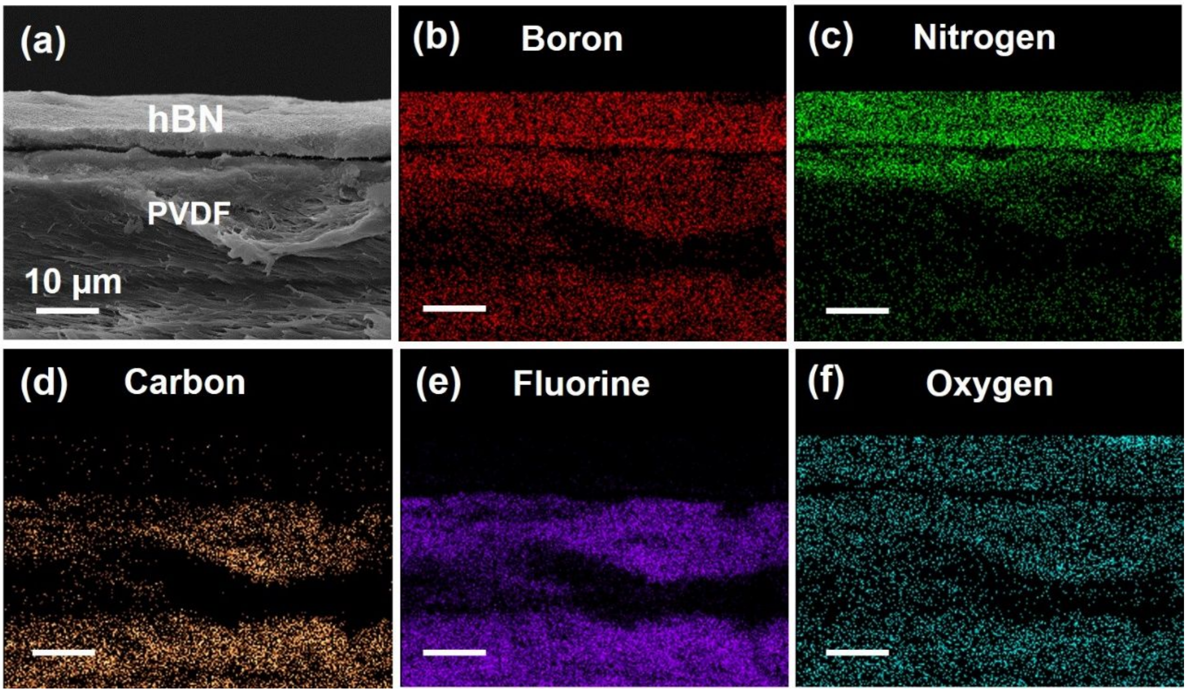

Figure S5. (a) Cross-sectional SEM image of hBN membrane with their corresponding elemental mapping showing (b) boron, (c) nitrogen, (d) carbon, (e) fluorine, and (f) oxygen. It is clearly seen that hBN nanosheets mostly assembly on a PVDF filter.

6. PXRD patterns of hBN membranes

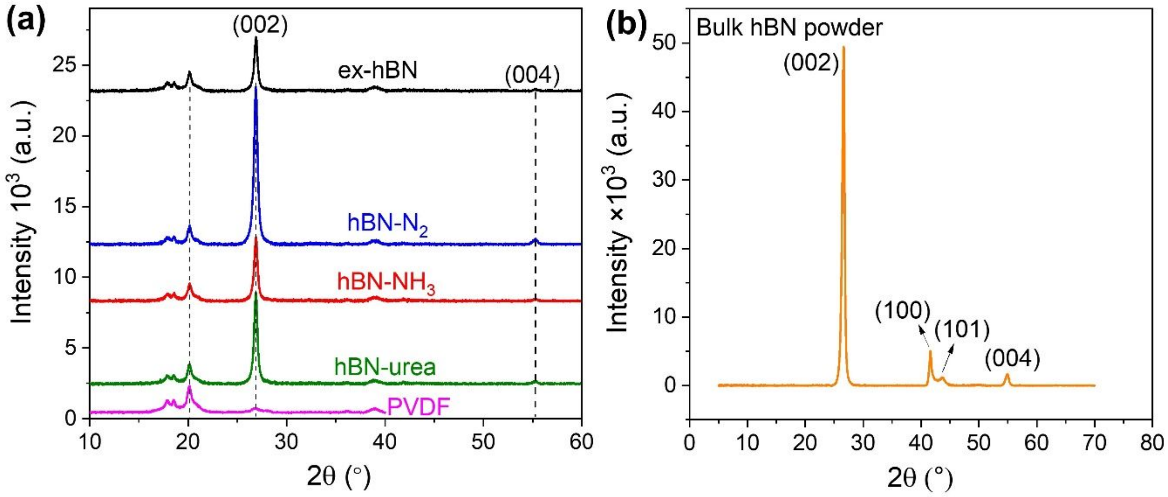

Figure S6. (a) Full PXRD pattern of each hBN membrane on a PVDF filter showing the characteristic (002) peak of laminar hBN membranes. (b) PXRD pattern of bulk hBN powder showing typical characteristic (002), (100), (101), and (004) peaks.

7. FTIR spectra of hBN samples

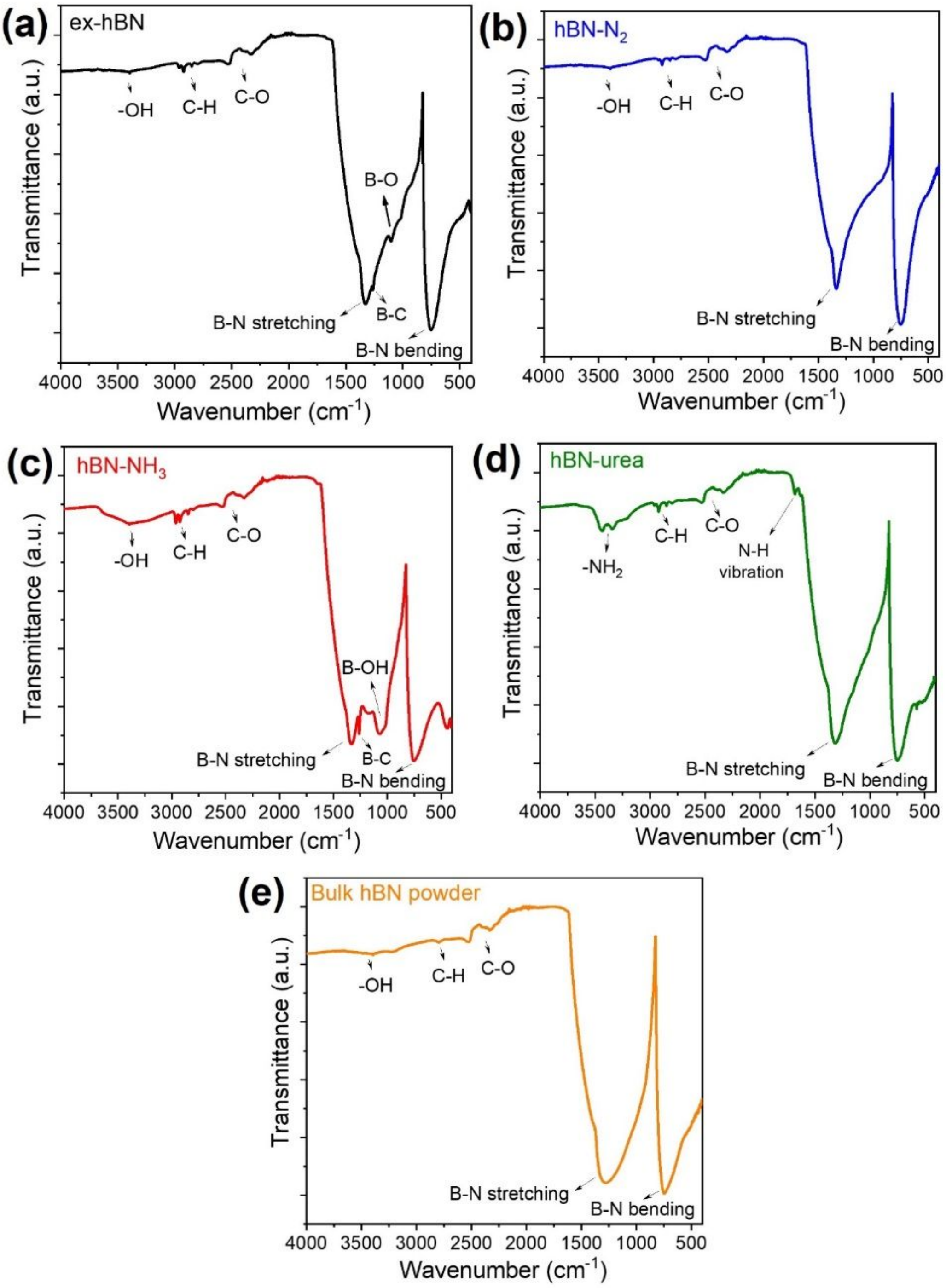

Figure S7. FTIR spectra of (a) ex-hBN, (b) hBN-N_2, (c) hBN-NH_3, (d) hBN-urea, and (e) bulk hBN powder.

8. XPS analysis of hBN samples

Table S1. The atomic percentage of each element on hBN samples as well as B:N, B:C, and B:O ratios.

**Elements**	**ex-hBN**	**hBN-N_2**	**hBN-NH_3**	**hBN-urea**	**Bulk hBN**
**B (at%)**	39.08	41.44	41.44	40.59	44.62
**N (at%)**	30.51	33.33	33.57	34.77	38.91
**C (at%)**	22.73	20.23	18.52	20.16	10.87
**O (at%)**	7.69	5.01	6.47	4.48	5.61
**B:N ratio**	1.28	1.24	1.23	1.17	1.15
**B:C ratio**	1.72	2.05	2.24	2.01	4.10
**B:O ratio**	5.08	8.27	6.40	9.06	7.95

Note the atomic percentage was obtained from XPS analysis.

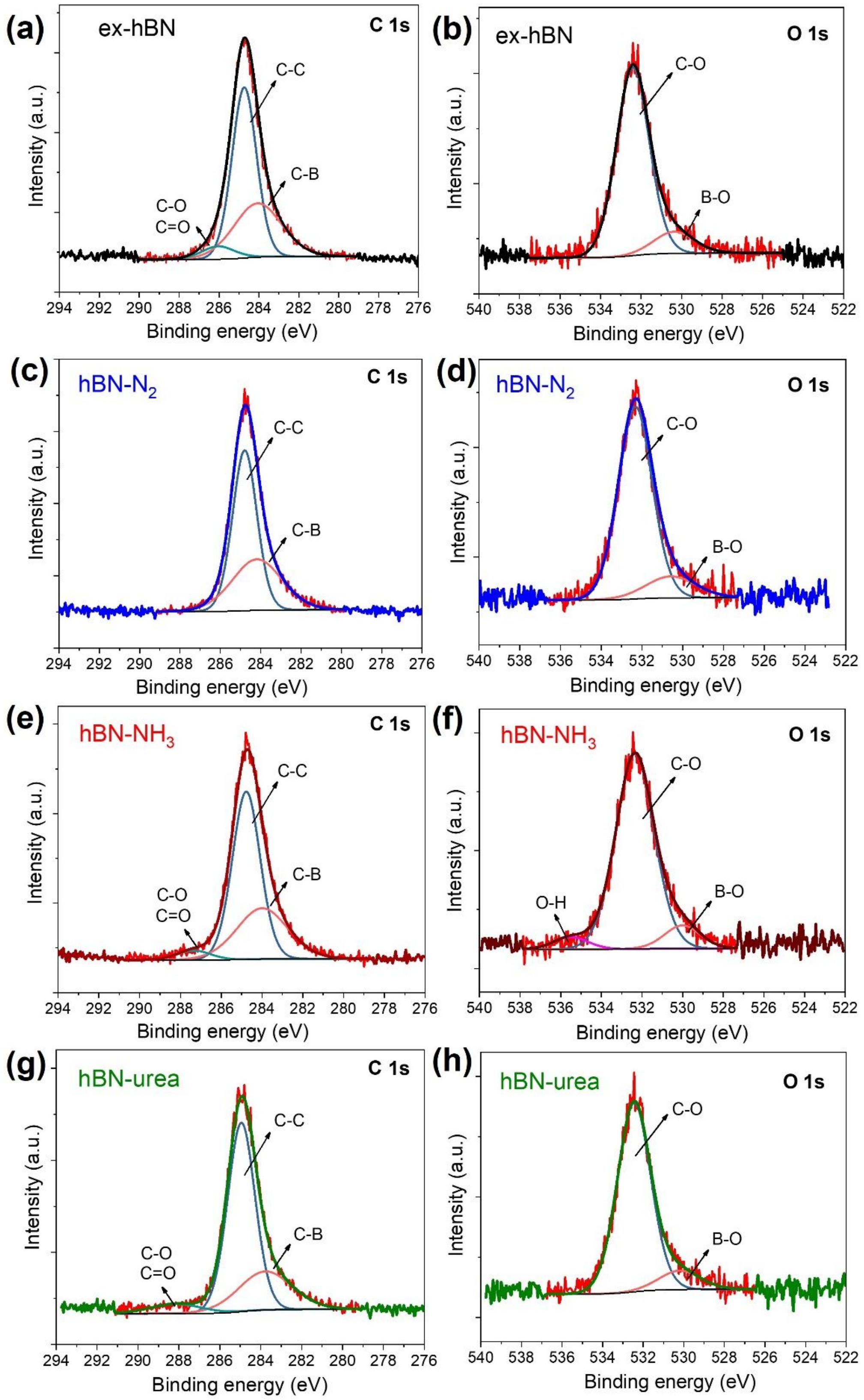

Figure S8. High-resolution XPS spectra of C 1s and O 1s peaks with their fitting curves for (a, b) ex-hBN, (c, d) hBN-N_2, (e, f) hBN-NH_3, (g, h) hBN-urea.

9. Comparison of literature value of 2D materials-based membranes for dye removal

Table S2. Dimensional size of charged dye molecules used as dye removal applications

Molecule	Dimensional size
Acid orange 7 (AO7)	5.4 × 10.0 × 15.7 Å (ref[1])
Methylene blue (MB)	3.3 × 7.6 × 17.0 Å (ref[2])

Table S3. Comparison of literature values of 2D materials-based membranes for dye removal.

Membranes (Test mode)	[c] Positively charged dye		[d] Negatively charged dye		Ref.
	**Rejection (%)**	**Water permeance (LMH/bar)**	**Rejection (%)**	**Water permeance (LMH/bar)**	
Porous MoS_2 (Dead-end)	~97 (MB) (very low feed)	N/A	~90 (MO) (very low feed)	N/A	3
hBN (Dead-end)	-	-	96.5 (AO7) (175 ppm feed)	N/A	4
[a] BNNS-20 (Dead-end)	~87 (MB) (30 ppm feed)	N/A	~98 (CR) (200 ppm feed)	N/A	5
BN membrane (Dead-end)	~99 (MB) (8.6 ppm feed)	N/A	~98 (MO) (16 ppm feed)	N/A	6
[b] BN-g-PMMA (Dead-end)	~95 (MB) (10 ppm feed)	N/A	~97 (AV7) (10 ppm feed)	N/A	7
Janus membrane/WS_2 (Forward osmosis)	~92 (RhB) (5 ppm feed)	0.069	-	-	8
Graphene (Forward osmosis)	97.9 (MB) (30 ppm feed)	0.0042	99.1 (AO7) (30 ppm feed)	0.0048	9
**ex-hBN (Forward osmosis)**	**93.4 (MB) (30 ppm feed)**	**0.05**	**97.2 (AO7) (30 ppm feed)**	**0.057**	**This work**
**hBN-N_2 (Forward osmosis)**	**96.8 (MB) (30 ppm feed)**	**0.043**	**98.1 (AO7) (30 ppm feed)**	**0.043**	**This work**
**hBN-urea (Forward osmosis)**	**93.8 (MB) (30 ppm feed)**	**0.028**	**97.5 (AO7) (30 ppm feed)**	**0.05**	**This work**
**hBN-NH_3 (Forward osmosis)**	**94.9 (MB) (30 ppm feed)**	**0.043**	**98.2 (AO7) (30 ppm feed)**	**0.036**	**This work**

[a] BNNS: boron nitride nanosheet membrane; [b] BN-g-PMMA : boron nitride grafted PMMA

[c] Positively charged dyes, MB: methylene blue; RhB: Rhodamine B.

[d] Negatively charged dyes, AO7: acid orange 7; MO: methyl orange; CR: Congo red; AV7: acid violet 7.

10. Supporting references

(1) Yoon, S.; Calvo, J. J.; So, M. C. Removal of Acid Orange 7 from Aqueous Solution by Metal-Organic Frameworks. *Crystals* **2019**, *9* (1), 17. DOI: 10.3390/cryst9010017.
(2) Arias, M.; López, E.; Nuñez, A.; Rubinos, D.; Soto, B.; Barral, M. T.; Díaz-Fierros, F. Adsorption of Methylene Blue by Red Mud, An Oxide- Rich Byproduct of Bauxite Refining. In *Effect of Mineral-Organic-Microorganism Interactions on Soil and Freshwater Environments*, Berthelin, J., Huang, P. M., Bollag, J. M., Andreux, F. Eds.; Springer US, 1999; pp 361-365.
(3) Sapkota, B.; Liang, W.; VahidMohammadi, A.; Karnik, R.; Noy, A.; Wanunu, M. High Permeability Sub-Nanometre Sieve Composite MoS_2 Membranes. *Nat. Commun.* **2020**, *11* (1), 2747. DOI: 10.1038/s41467-020-16577-y.
(4) Pendse, A.; Cetindag, S.; Lin, M.-H.; Rackovic, A.; Debbarma, R.; Almassi, S.; Chaplin, B. P.; Berry, V.; Shan, J. W.; Kim, S. Charged Layered Boron Nitride-Nanoflake Membranes for Efficient Ion Separation and Water Purification. *Small* **2019**, *15* (49), 1904590. DOI: 10.1002/smll.201904590.
(5) Wang, Z.; Zhu, Y.; Ji, D.; Li, Z.; Yu, H. Scalable Exfoliation and High-Efficiency Separation Membrane of Boron Nitride Nanosheets. *ChemistrySelect* **2020**, *5* (12), 3567-3573. DOI: 10.1002/slct.202000622.
(6) García Doménech, N.; Purcell-Milton, F.; Sanz Arjona, A.; Casasín García, M.-L.; Ward, M.; Cabré, M. B.; Rafferty, A.; McKelvey, K.; Dunne, P.; Gun'ko, Y. K. High-Performance Boron Nitride-Based Membranes for Water Purification. *Nanomaterials* **2022**, *12* (3), 473. DOI: 10.3390/nano12030473.
(7) Samantaray, P. K.; Sen Gupta, R.; Bose, S. Self-Assembly in "Matrix-Free" Functionalized Boron Nitride Sheets as Free-Standing Thin Film Sieves for Stable Forward Osmosis and Robust Dye Removal Applications. *Adv. Sustainable Syst.* **2023**, *7* (3), 2200385. DOI: 10.1002/adsu.202200385.
(8) Bermúdez-Morales, L.; Lizardi, L.; Santiago-Martoral, L.; Cruz-Tato, P.; Nicolau, E. Zwitterionic and Photocatalytic Janus Membrane Based on Diethylamine N-Oxide Group and WS_2 Nanosheets for Dye Removal in Forward Osmosis. *ACS Appl. Eng. Mater.* **2023**, *1* (8), 2236-2246. DOI: 10.1021/acsaenm.3c00296.
(9) Paechotrattanakul, P.; Jitapunkul, K.; Iamprasertkun, P.; Srinoi, P.; Sirisaksoontorn, W.; Hirunpinyopas, W. Ultrahigh Stable Laminar Graphene Membranes for Effective Ionic and Molecular Nanofiltration with a Machine Learning-Assisted Study. *Nanoscale* **2023**, *15* (19), 8716-8729. DOI: 10.1039/D2NR06969E.
